# Supplementary material for: Structure of the TFIIIC subcomplex τA provides insights into RNA polymerase III pre-initiation complex formation
Source: Nat Commun. 2020 Sep 30;11:4905. doi: 10.1038/s41467-020-18707-y (PMC7528018; doi:10.1038/s41467-020-18707-y)
Supplement: Supplementary file 1 — Supplementary Information [file 41467_2020_18707_MOESM1_ESM.pdf]

Supplementary Information

Structure of the TFIIIC subcomplex τA provides insights into RNA polymerase III pre-initiation complex formation

Vorländer et al.

SUPPLEMENTARY FIGURES

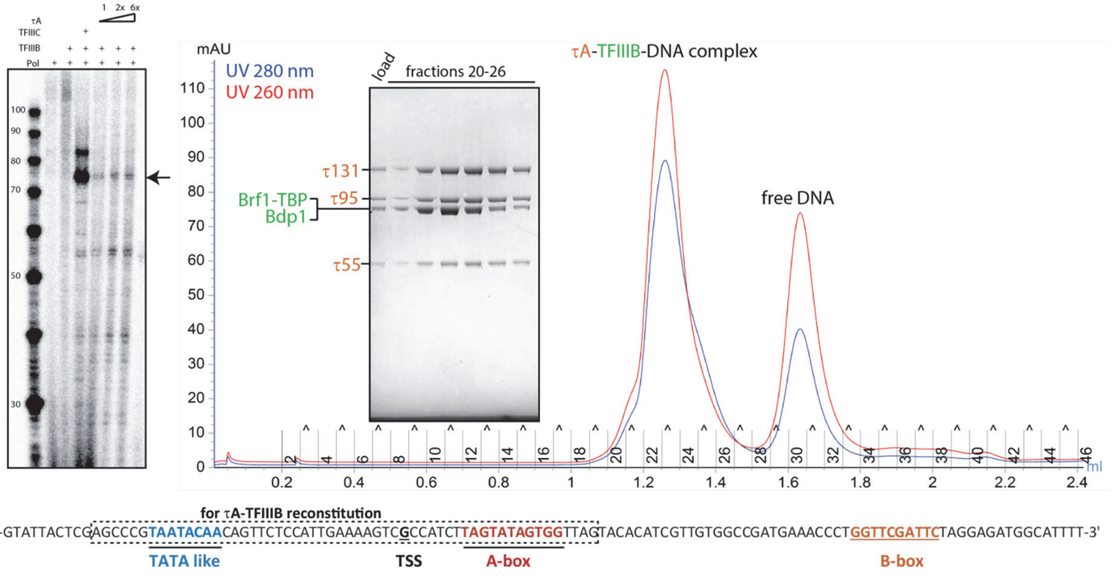

Supplementary Figure 1. τA stimulates transcription from a TFIIIC-dependent promoter at low levels and binds TFIIIB. *In vitro* transcription assay, comparing full-length TFIIIC and τA. Only the sequence of the NT DNA strand used for the transcription assay is shown below, with the sequence used for reconstitution of τA-TFIIIB marked with a dotted box. Right: SEC profile on a Superdex 200 column and SDS-PAGE of indicated fractions show that τA binds TFIIIB. Source data are provided as a Source Data file.

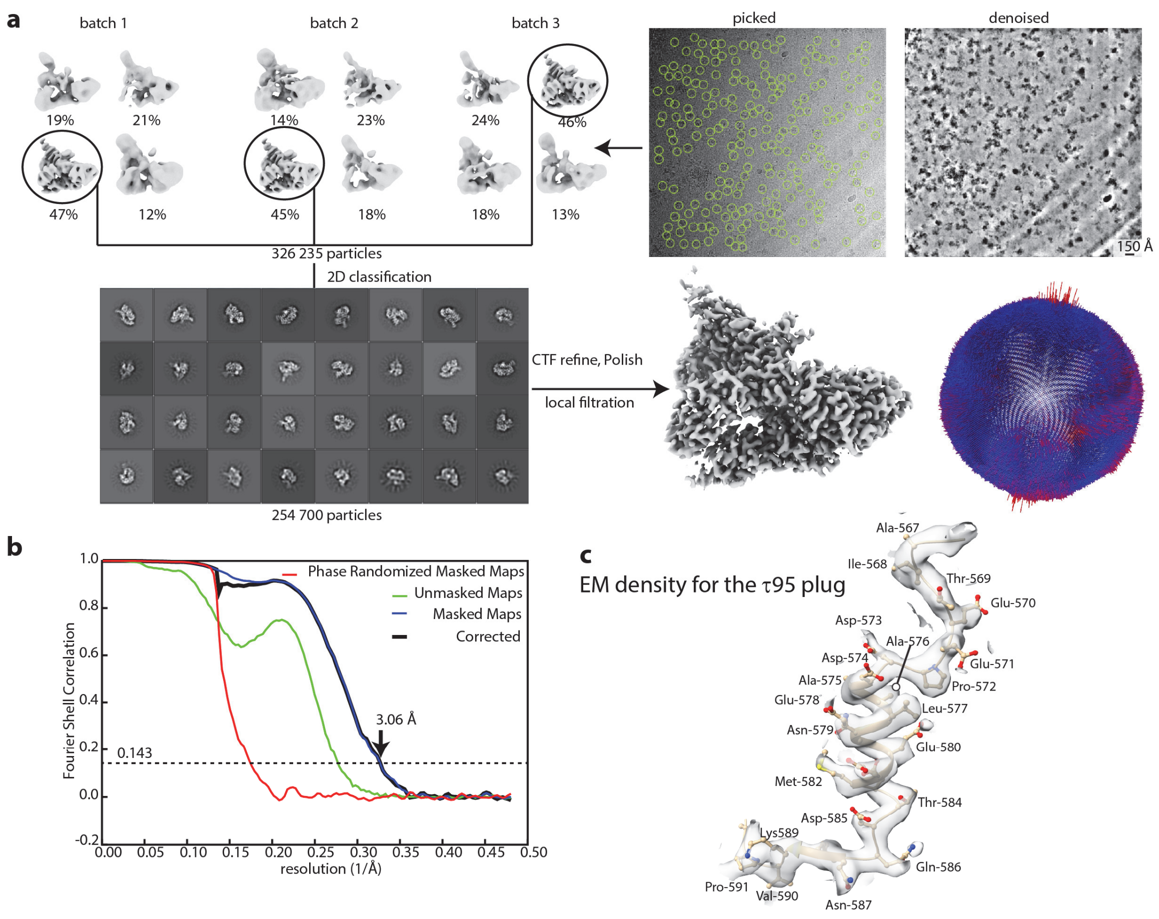

Supplementary Figure 2. Cryo-EM structure determination of the τA subcomplex. a, Cryo-EM particle classification and refinement. 702,583 particles were picked and divided into three batches. Each batch was subjected to 3D classification in RELION, using a 40 Å lowpass filtered negative stain map of the same complex as a reference. The best class of each batch was selected, particles from all batches were pooled and further cleaned through 2D classification in RELION (setting the skip setting the "Ignore CTFs until first peak" option to "yes"). 254,700 particles were selected and refined. Two rounds of CTF refinement and particle polishing in RELION yielded a map with 3.06 Å resolution. The final map is shown after sharpening with a B-factor of -55 A^2 and local filtering. The angular distribution of particle views is plotted onto a sphere. The height of the cylinders is proportional to the number of views from that angle. Red cylinders are overrepresented views. **b**, FSC plot as calculated by the *relion_postprocess* program. **c,** EM density of the acidic plug with residues labeled. Note that density for carboxylate groups are frequently absent in cryo-EM maps due to radiation damage.

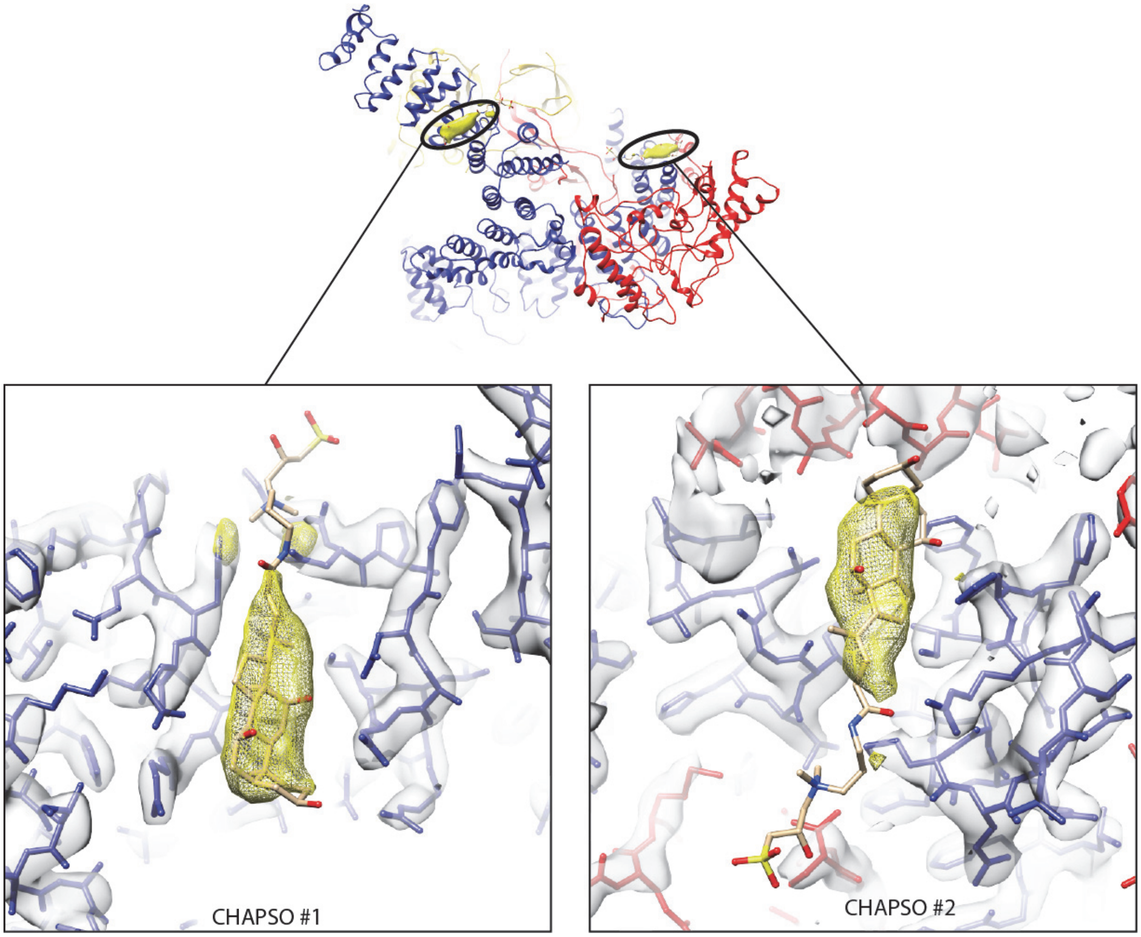

Supplementary Figure 3. The EM map shows two ordered molecules of the detergent CHAPSO bound to τA. Protein density is shown as a grey transparent surface, and CHAPSO density is shown as a yellow mesh. One molecule ('CHAPSO #1') is located between TPR4 and 5 in τ131. The second molecule is located between the unassigned helical τ95 density and the C-terminal TPR array of τ131. The CHAPSO molecules were not included in the deposited PDB file because their orientation cannot be unambiguously assigned.

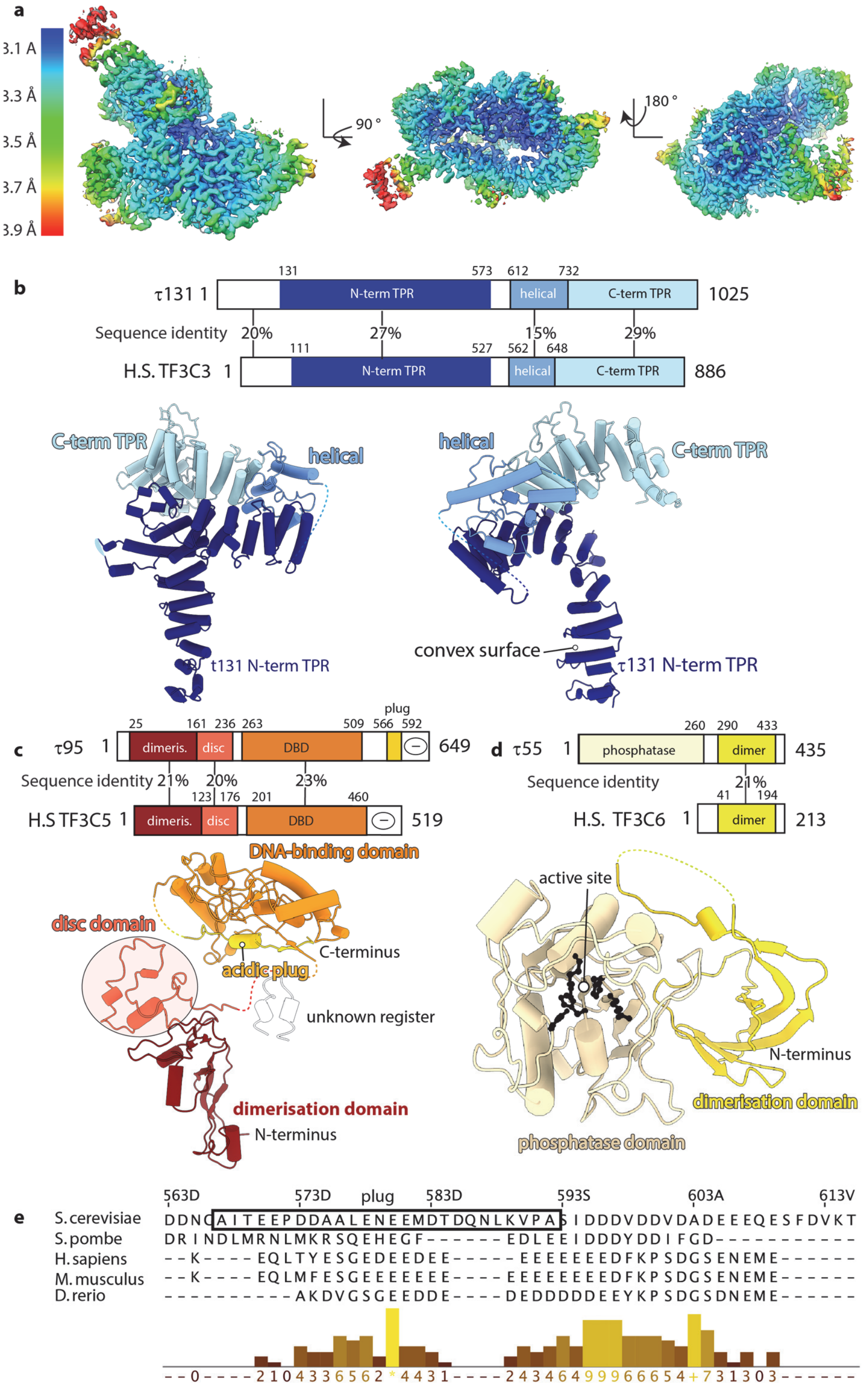

Supplementary Figure 4. Cryo-EM structure of the τA subcomplex. **a**, cryo-EM density colored by local resolution.. **b-d,** Structures of τA subunits τ131, τ95 and τ55 with domain annotation. Predicted domain boundaries of human homologs and the sequenced identity between corresponding domains are indicated. Protein domains are coloured as in the corresponding domain diagrams, with τ131 in shades of blue, τ95 in red and orange, and τ55 in wheat and yellow. **e,** Multiple sequence alignment of τ95 homologs, showing the region around the acidic plug. The numbering above corresponds to the *S. cerevisiae* residues. The acidic plug does not appear to be conserved outside *cerevisiae* species.

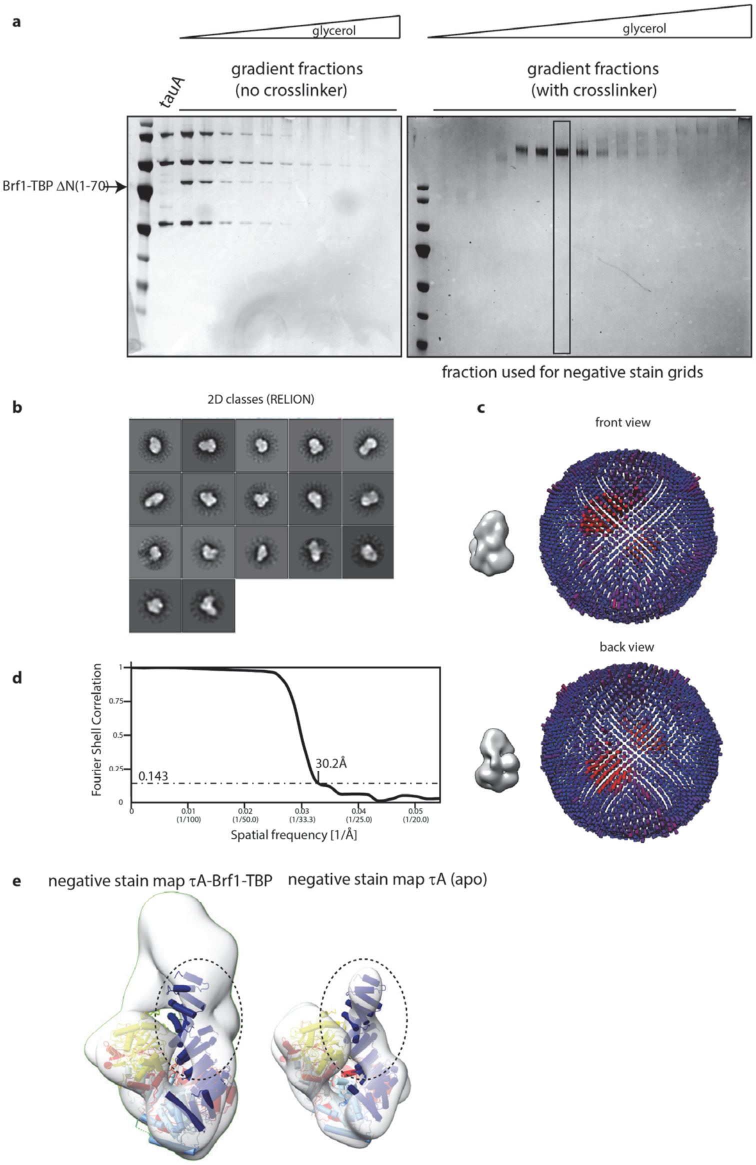

Supplementary Figure 5. Negative stain structure determination of the τA-Brf1-TBP complex. a, SDS-PAGE analysis of fractions of τA-Brf1-TBP purified over a glycerol gradient in absence (left) or presence (right) of 0.1% glutaraldehyde in the heavy buffer. **b**, Selected negative stain 2D class averages (computed in RELION). **c**, Two views of the final negative stain map, and the angular distribution coverage. **d**, FSC plot of the two independent half maps and resolution estimate according to the 0.143 cutoff criterion. **e,** Comparison of the fit of the τA cryo-EM structure to negative stain maps of Brf1-TBP-τA and to τA only. The dashed circle highlights the fit of the N-term TPR array to the density, indicating that in the Brf1-TBP bound state this portion might be shifted.

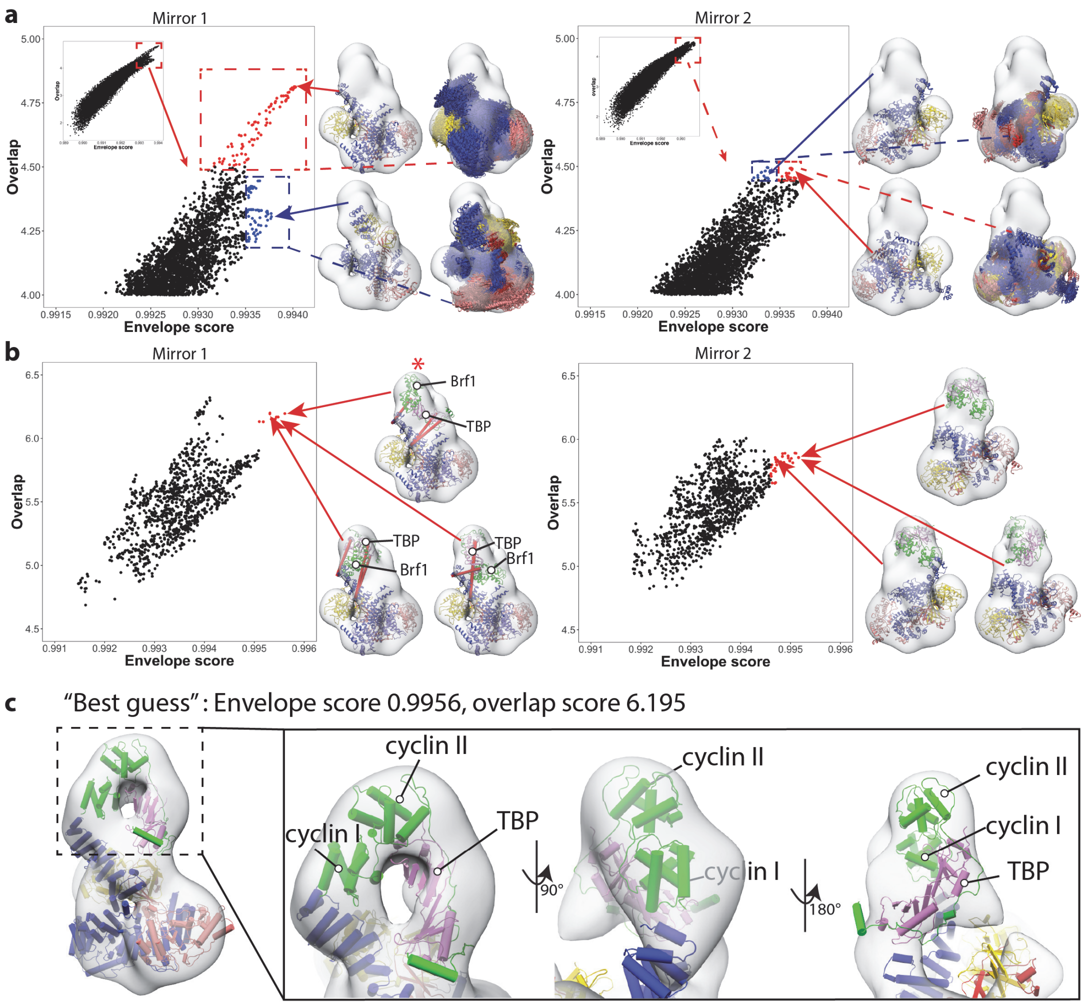

Supplementary Figure 6. Fitting of τA and Brf1-TBP to the negative stain EM map. a, τA structure fits to the mirror map 1 with better scores than to the mirror map 2. The plots show the distribution of scores for all fits (inset) and the top fits (main plot) as described in Methods. For each mirror map, selected best scoring fits are colored blue and red. The structures corresponding to these fits are shown for a representative fit and for all fits from each subgroup overlaid. **b,** Models combining alternative fits of both τA and Brf1-TBP also give better scores for the mirror map 1, with top fits according to the overlap and envelope scores indicated in red. The top fit and two fits selected from the other top fits are shown within the EM maps. Red bars on the structures represent the three crosslinks between Brf1-TBP and τA that could be mapped to structurally resolved regions of the structures. **c,** Depiction of the *–marked model shown in b,. The inset shows a close-up of the Brf1-TBP fit, indicating that the additional density fits can be explained with the known structure of the Brf1-TBP complex.

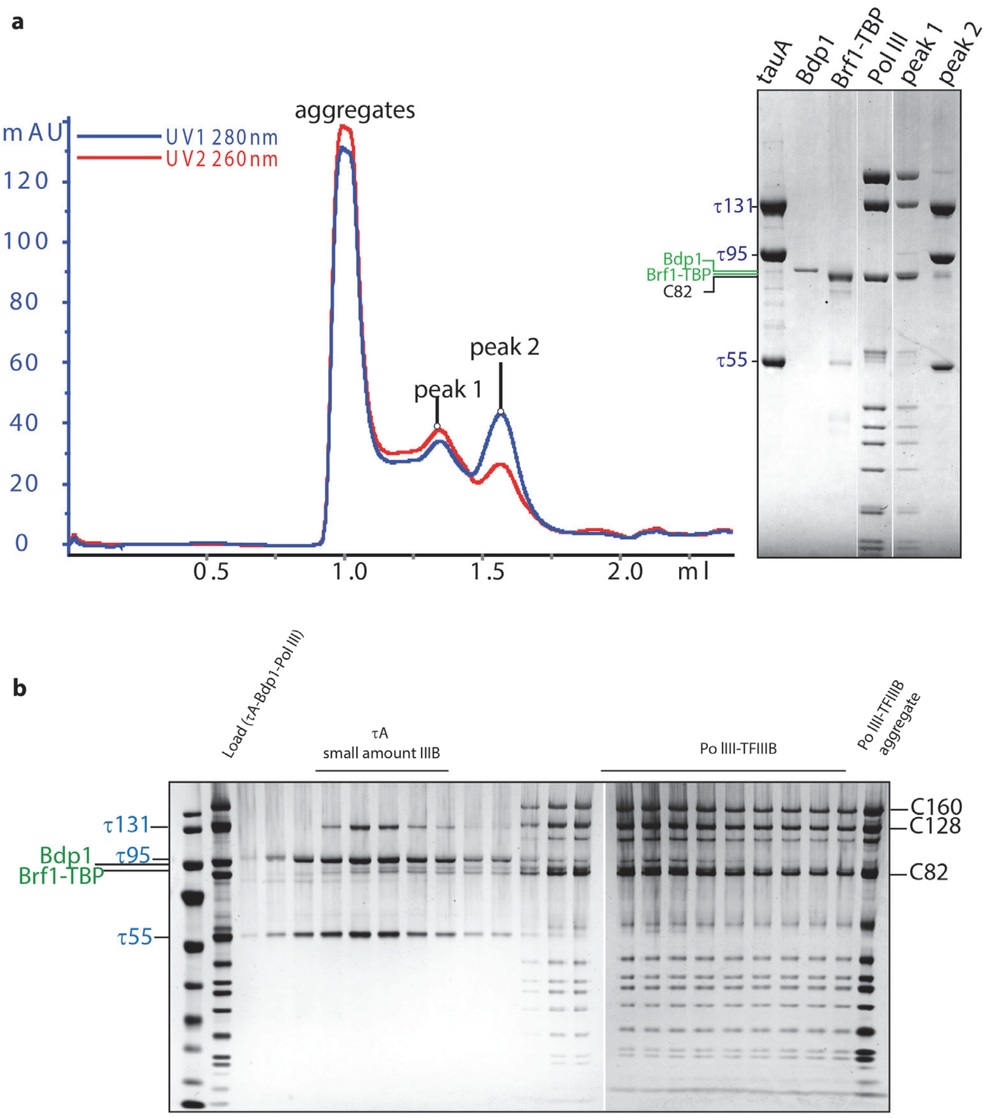

Supplementary Figure 7. τA is displaced from TFIIIB when Pol III is added. a, Size exclusion chromatogram (left) and SDS-PAGE showing purified protein and peak fractions as indicated. The gel image on the right shows lanes cropped form the same gel. **b**, The same sample was purified over a 15%-45% glycerol gradient and fractions were analyzed by SDS-PAGE and silver staining. Lane 2 shows a sample of Pol III, τA,Pol III and Brf1-TBP mixed and loaded as a reference. τA elutes separately from Pol III-TFIIIB. Source data are provided as a Source Data file.

Supplementary Table 1

Cryo-EM data collection, refinement and validation statistics

**Data collection and processing**	
Magnification	130,000
Voltage (kV)	300
Electron exposure (e–/Å^2)	48.7
Defocus range (μm)	-0.5 to -2
Pixel size (Å)	1.041
Symmetry imposed	C1
Initial particle images (no.)	704 446
Final particle images (no.)	254 700 (36%)
Map resolution (Å)	3.1
FSC threshold	0.143
Map resolution range (Å)	2.9-4.5
**Refinement**	
Model resolution (Å)	3.3
Map sharpening *B* factor (Å^2)	-55
**Model composition**	
Non-hydrogen atoms	13520
Protein residues	1657
Ligands	0
***B* factors (Å^2)**	
Protein	68
Ligand	n/a
**R.m.s. deviations**	
Bond lengths (Å)	0.014
Bond angles (°)	1.145
**Validation**	
MolProbity score	1.31
Clashscore	1.89
Poor rotamers (%)	0.7
**Ramachandran plot**	
Favored (%)	94.8
Allowed (%)	4.6
Disallowed (%)	0.01
