# Supplementary material for: Structure of the TFIIIC subcomplex τA provides insights into RNA polymerase III pre-initiation complex formation
Source: Nat Commun. 2020 Sep 30;11:4905. doi: 10.1038/s41467-020-18707-y (PMC7528018; doi:10.1038/s41467-020-18707-y)

Figure 2c

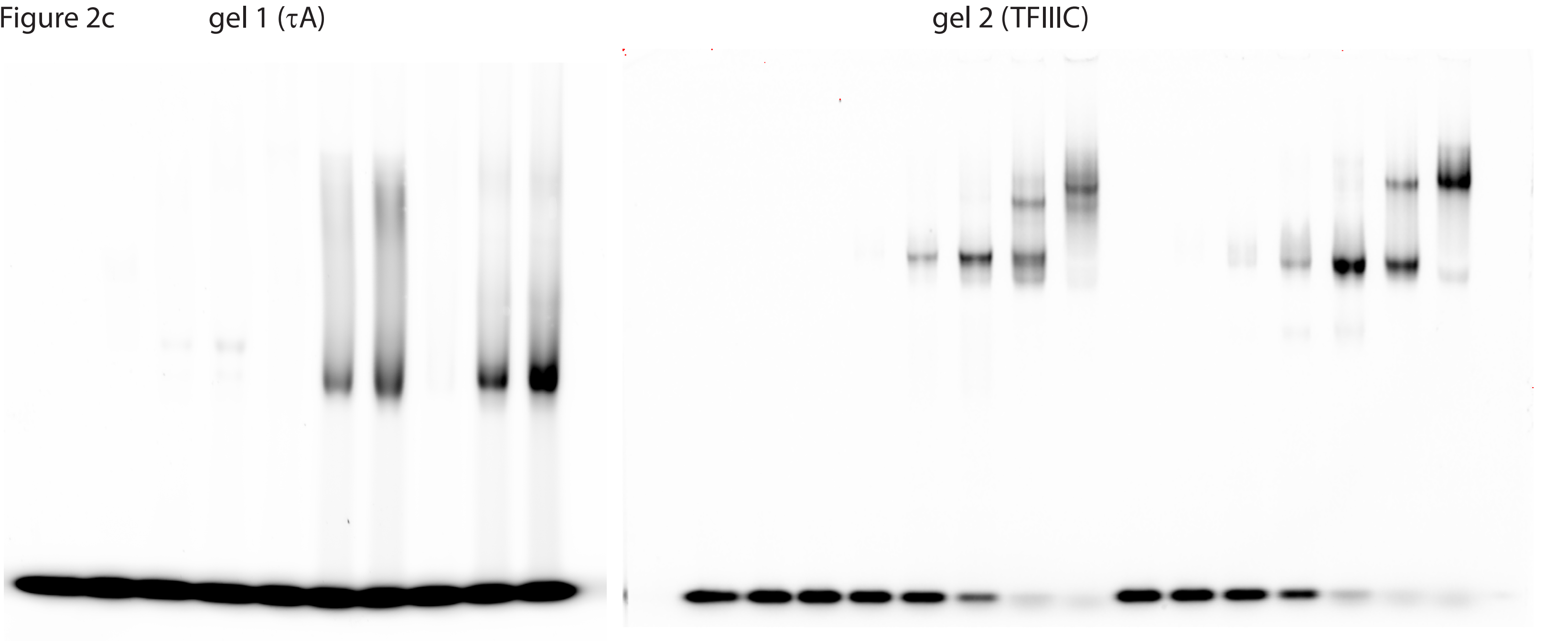

Figure 2d

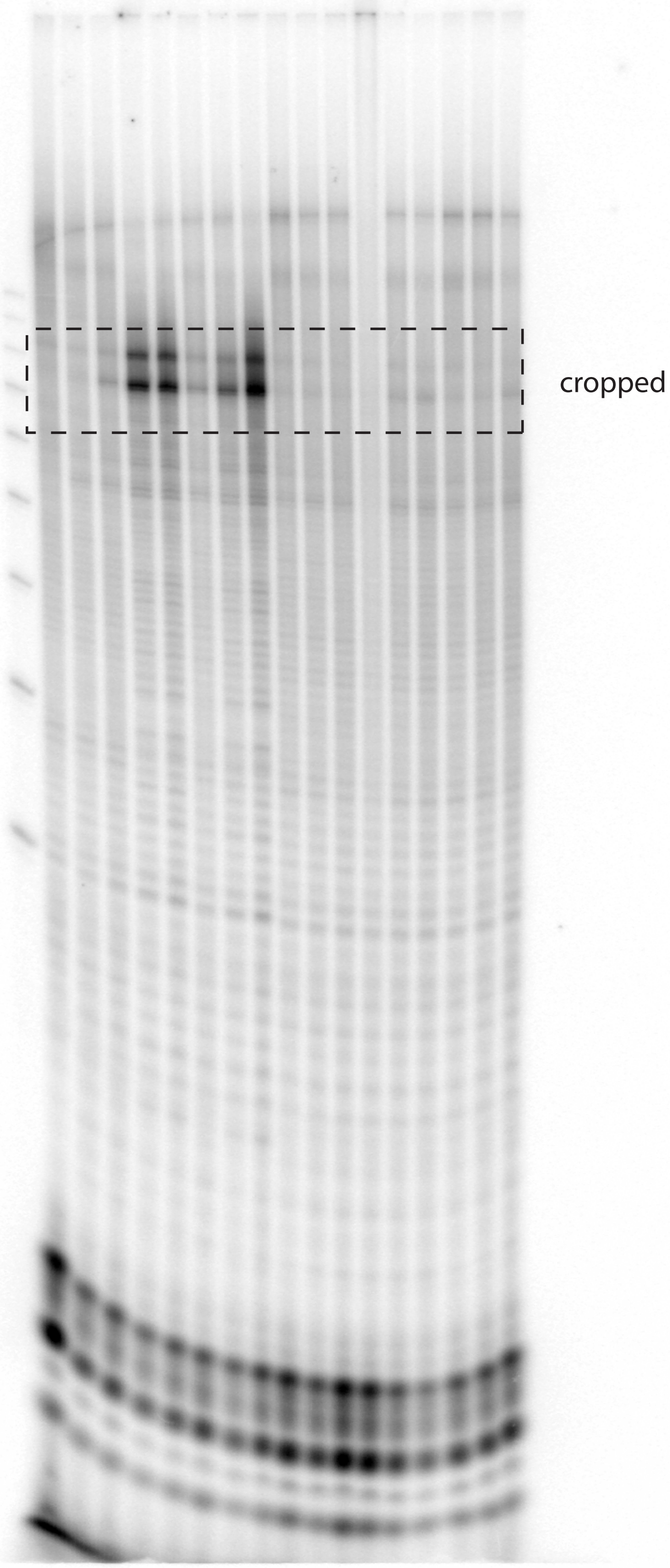

Extended Data Figure 1

gel 1

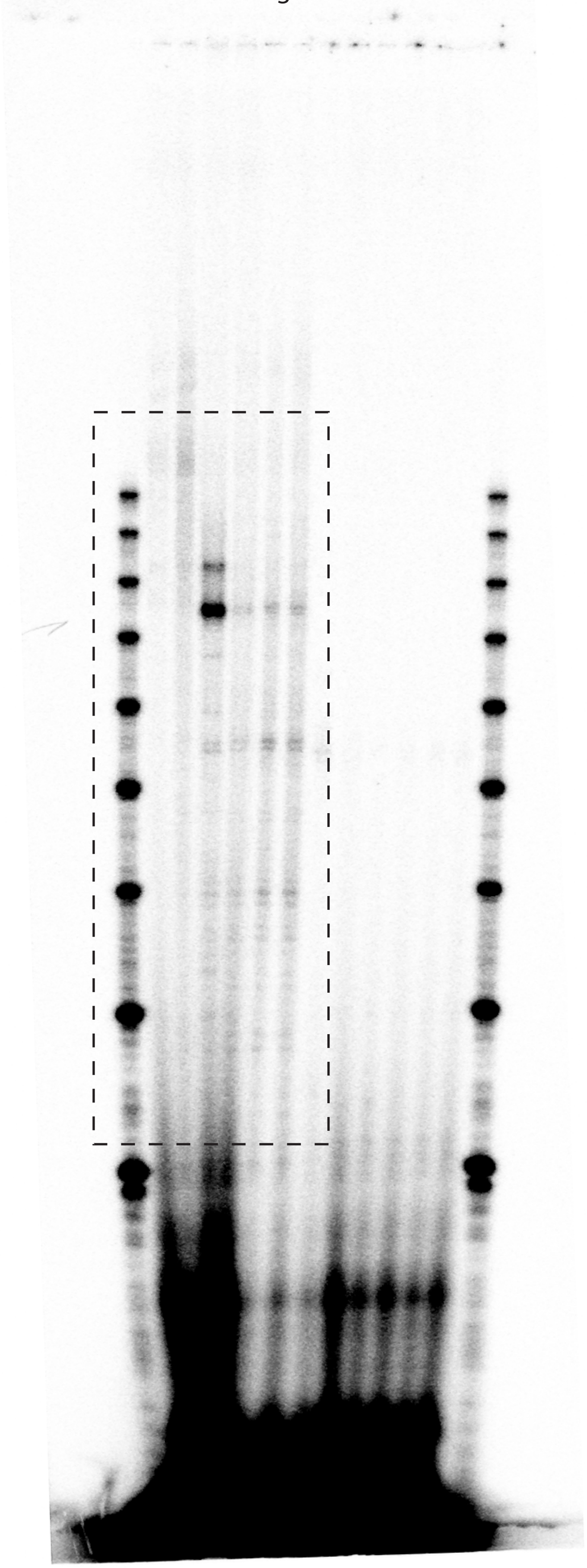

gel 2

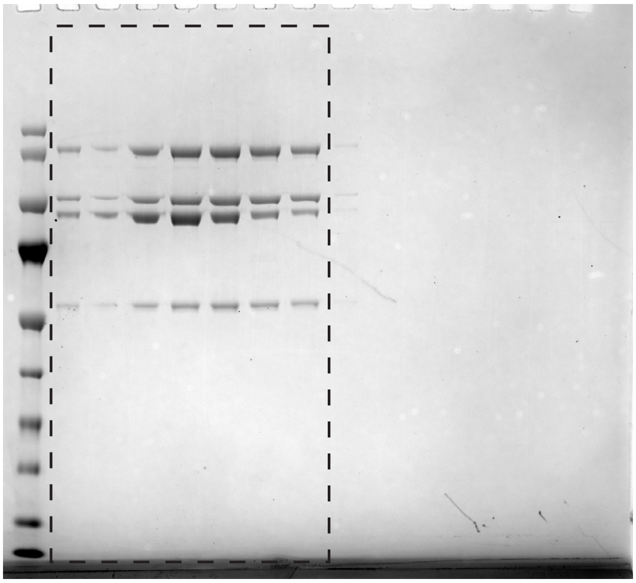

Extended Data Fig 7a

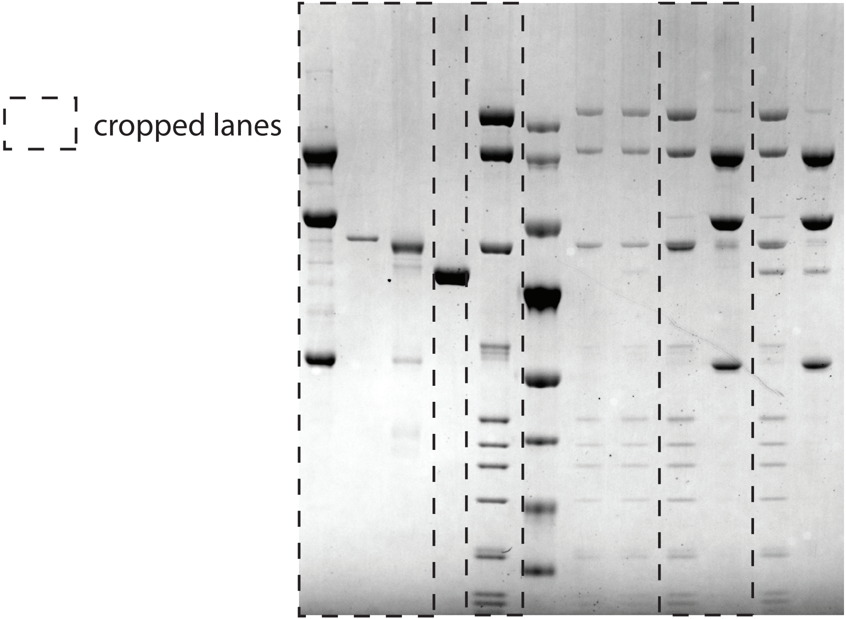

Extended Data Figure 7b

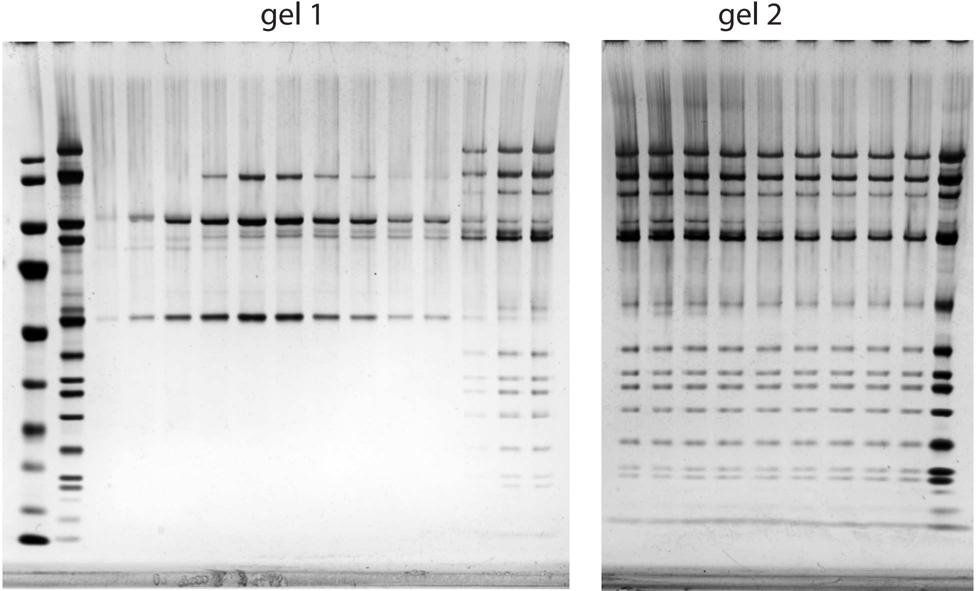

Supplement: Supplementary file 7 — Source data [file 41467_2020_18707_MOESM7_ESM.pdf]
